# Test-retest variability of adenosine A_2A_ binding in the human brain with ^11^C-TMSX and PET

**DOI:** 10.1186/s13550-014-0076-9

**Published:** 2014-12-29

**Authors:** Mika Naganawa, Masahiro Mishina, Muneyuki Sakata, Keiichi Oda, Mikio Hiura, Kenji Ishii, Kiichi Ishiwata

**Affiliations:** PET Center, Yale University School of Medicine, 801 Howard Avenue, New Haven, 06520-8048 CT USA; Research Team for Neuroimaging, Tokyo Metropolitan Institute of Gerontology, Tokyo, 173-0015 Japan; Department of Neurological Science, Graduate School of Medicine, Nippon Medical School, Tokyo, 113-0022 Japan; Department of Radiological Technology, Faculty of Health Sciences, Hokkaido University of Science, Hokkaido, 006-8585 Japan; Faculty of Sports and Health Studies, Hosei University, Tokyo, 194-0298 Japan

**Keywords:** Adenosine A2A receptor, Positron emission tomography, 11C-TMSX, Reproducibility

## Abstract

**Background:**

The goal of the present study was to evaluate the reproducibility of cerebral adenosine A_2A_ receptor (A_2A_R) quantification using ^11^C-TMSX and PET in a test-retest study.

**Methods:**

Five healthy volunteers were studied twice. The test-retest variability was assessed for distribution volume (*V*_T_) and binding potential relative to non-displaceable uptake (BP_ND_) based on either metabolite-corrected arterial blood sampling or a reference region. The cerebral cortex and centrum semiovale were used as candidate reference regions.

**Results:**

Test-retest variability of *V*_T_ was good in all regions (6% to 13%). In the putamen, BP_ND_ using the centrum semiovale displayed a lower test-retest variability (3%) than that of BP_ND_ using the cerebral cortex as a reference region (5%). The noninvasive method showed a higher or similar level of test-retest reproducibility compared to the invasive method.

**Conclusions:**

Binding reproducibility is sufficient to use ^11^C-TMSX as a tool to measure the change in A_2A_R in the human brain.

**Electronic supplementary material:**

The online version of this article (doi:10.1186/s13550-014-0076-9) contains supplementary material, which is available to authorized users.

## Background

The regional cerebral binding of adenosine A_2A_ receptor (A_2A_R) antagonists, [7-methyl-^11^C]-(*E*)-8-(3,4,5-trimethoxystyryl)-1,3,7-trimethylxanthine (^11^C-TMSX) [1] and ^11^C-KW-6002 [2],[3], were quantitatively investigated *in vivo* in healthy human. ^11^C-TMSX has been evaluated in human brain studies in not only healthy human controls [4]-[7] but also in drug-naïve Parkinson's disease patients before and after therapy [8]. An aging effect on A_2A_R was also evaluated using ^11^C-TMSX PET [9]. Several outcome measures, such as distribution volume (*V*_T_), distribution volume ratio (DVR), and binding potential (BP_ND_), can be used to detect changes of A_2A_R binding due to disease progression or therapeutic treatment. The reproducibility of these measures is important to conduct studies to detect change in A_2A_R. Given that A_2A_R distribution is heterogeneous, with only a very small amount of extrastriatal-specific binding, either the frontal cortex in the rat [3], the cerebellum in the monkey [10],[11], or centrum semiovale [7] or cerebral cortex [8],[9] in human, was used as a reference region to estimate non-displaceable binding of ^11^C-TMSX. Since only a few *postmortem* human brain studies and blocking studies are available, it is not clear which region is a suitable reference region.

The aim of this paper was to assess the test-retest reproducibility of PET outcome measures, *V*_T_ and BP_ND_, with the centrum semiovale and cerebral cortex as candidate reference regions.

## Methods

### Human subjects

All studies were performed under a protocol approved by the Ethics Committee of the Tokyo Metropolitan Institute of Gerontology. Five healthy, male subjects participated in this study (mean age ± SD, 22.4 ± 2.6 years old, range: 21 to 27 years old). Subjects were all right-handed and screened for history of neurological, psychiatric, and physical diseases. All subjects did not have a history of alcoholism and not on any medications to affect brain function. Caffeine intake was not allowed for at least 12 h prior to PET scanning. Written informed consent was obtained from all subjects after receiving an explanation of the study. Magnetic resonance (MR) images were acquired on all subjects to eliminate those with any brain abnormalities and to place regions of interest (ROIs) on PET images. The MR imaging was conducted with three-dimensional spoiled gradient-recalled echo (SPGR) imaging on a SIGNA 1.5 Tesla machine (General Electric, Waukesha, WI, USA) [6].

### Radiochemistry

Radiosynthesis of ^11^C-TMSX followed the literature procedure [12]. All procedures were conducted under dim light to prevent photoisomerization of ^11^C-TMSX. The radiochemical purity of ^11^C-TMSX was >99%.

### PET acquisition

Each subject underwent two ^11^C-TMSX brain PET scans on two different days, and time of scanning was identical for test and retest scans of each individual subject, in order to remove the influence of circadian rhythm. The inter-scan interval was 28 to 35 days. Dynamic PET images were acquired in the Positron Medical Center, Tokyo Metropolitan Institute of Gerontology with the SET-2400 W PET scanner (Shimadzu, Kyoto, Japan), which acquires 63 slices (3.125-mm slice separation) with a spatial resolution of 4.4 mm full width at half maximum (FWHM) and a z-axis resolution of 6.5 mm FWHM [13]. Prior to the scan, a 5-min ^68^Ga/^68^Ge transmission scan was conducted for attenuation correction. ^11^C-TMSX was injected intravenously over 60 s. Emission data were collected in two-dimensional mode for 1 h in 27 frames of increasing duration (6 × 10 s; 3 × 30 s; 5 × 1 min; 5 × 2.5 min; 8 × 5 min). Head movement was minimized with an air cushion. The dynamic images were reconstructed by the filtered back-projection method using a Butterworth filter (second-order low-pass filter, cutoff frequency was 1.25 cycles/cm) with corrections for scatter and randoms.

### Input function measurement

In advance of each scan, an arterial catheter was inserted into the radial artery for blood sampling. After radiotracer injection, arterial blood samples were manually collected every 10 s for the first 2 min and thereafter at longer intervals, 2.25, 2.5, 3, 5, 7, 10, 15, 20, 30, 40, 50, and 60 min post-injection. A total of 24 samples were obtained per scan. Whole blood and plasma were counted in a cross-calibrated well-type gamma-counter (BSS-1, Shimadzu, Kyoto, Japan). An additional venous blood sample was taken before ^11^C-TMSX administration, which was used for the *in vitro* assessment of the fraction of ^11^C-TMSX in plasma bound to plasma proteins (*f*_P_). Arterial blood sampling was not available in one subject. Thus, a total of five subjects were included in reference region analyses and four subjects were also analyzed using plasma data.

### Plasma metabolite and protein binding analysis

The fraction of intact radioligand to total plasma activity was determined from blood samples collected at 3, 10, 20, 30, 40, and 60 min after injection by high-performance liquid chromatography (HPLC). The blood was centrifuged at × 7,000 *g* for 1 min at 4°C to obtain the plasma, which was denatured with an equivalent volume of acetonitrile in an ice-water bath. The suspension was centrifuged under the same conditions and divided into soluble and precipitable fractions. The precipitate was resuspended in 2 vol. of 50% aqueous acetonitrile followed by centrifugation. The recovery yield of the radioactivity in the two soluble fractions was 98.7%. Two soluble fractions were combined, and into this solution, an equivalent volume of a solution of 50-mM aqueous acetic acid and 50-mM aqueous sodium acetate (pH 4.5; 50/50, *v*/*v*) was added. After centrifugation of the samples as described above, the supernatant was loaded onto a Nova-Pak C8 column equipped in an RCM 8 × 10 module (8 mm diameter × 100 mm length; Millipore-Waters, Milford, MA, USA). The mobile phase was a mixture of acetonitrile, 50-mM aqueous acetic acid and 50-mM aqueous sodium acetate (pH 4.5; 4/3/3, *v*/*v*/*v*) at a flow rate of 2 mL/min. The elution profile was detected with a radioactivity monitor (FLO-ONE 150TR; Packard Instrument, Meriden, CT, USA). The retention time of ^11^C-TMSX was 6.2 min. The recovery in the eluate of the injected radioactivity was essentially quantitative. The six measured parent fractions were fitted by a sum of exponential functions. The metabolite-corrected plasma curve was generated as the product of the total plasma activity and the fitted parent fraction curve.

Individual *f*_P_ values were determined by ultrafiltration. Prior to administration of ^11^C-TMSX, approximately 6 mL of blood was taken from each subject. A reference blood sample was created by adding 22.9 ± 15.7 MBq (at the time of administration, range: 10.1 to 49.5 MBq of ^11^C-TMSX in approximately 60 μL to this blood sample and incubated for 10 min at 37°C). Following centrifugation (2,000 *g* at room temperature for 3 min), triplicates of 400 μL aliquots of plasma sample were pipetted into ultrafiltration tubes (Microcon-30, 30 kDa, Merck Millipore, Billerica, MA, USA), and centrifuged at room temperature (14 min at 14,000 *g*). The free fraction *f*_P_ was calculated as the ratio of activity in the ultrafiltrate to the total plasma. The amount of nonspecific binding of ^11^C-TMSX to the filter was also determined by applying the same procedure to a sample created by addition of ^11^C-TMSX to saline.

### Image analysis

Regions of interest were defined by manually drawing circles using the registered MR images as additional reference. The details are written in [6],[14]. Time-activity curves (TACs) were generated for eight ROIs: anterior putamen, posterior putamen, putamen, caudate head, thalamus, cerebellum, centrum semiovale, and cerebral cortex. The putamen ROI consists of the anterior and posterior putamen subregions. The cerebral cortex ROI included the frontal, temporal, and occipital cortices.

In the present study, the cerebral cortex and centrum semiovale were chosen as candidate reference regions. For ^11^C-TMSX kinetic analysis, the cerebellum was not used as a reference region, because A_2A_R binding in our previous human study [7] was higher in the cerebellum than in neocortical regions. In a previous human autoradiographic study [15], the density of A_2A_Rs in the frontal cortex was found to be low, as that in the temporal and occipital cortices.

### Outcome measures

The DVR has been used in our previous study on an aging effect of A_2A_R in human brain [8],[9]. In this study, the two additional outcome measures, *V*_T_ and BP_ND_, were estimated. The definition of the outcome measures is described in [16]. Regional TACs were analyzed using the Logan graphical analysis (LGA) with input function and reference tissue (two-parameter version) [17],[18] to estimate the outcome parameters of *V*_T_ and BP_ND_. Starting time (t*) was set to 10 min post-injection [7].

### Statistical analyses

The test-retest reproducibility was statistically evaluated according to the following three criteria: signed test-retest variability (TRV), absolute test-retest variability (aTRV), and intra-class correlation coefficient (ICC). TRV was calculated as the difference between the test and retest measurements, divided by the mean of the test and retest values (2 × (*p*_test_ − *p*_retest_)/(*p*_test_ + *p*_retest_)). aTRV was calculated as the absolute value of TRV (2 × |*p*_test_ − *p*_retest_|/(*p*_test_ + *p*_retest_)). TRV indicates whether there is a systematic trend between the test and retest scans. The test-retest reliability of the two parameter measurements was the ICC calculated using the following equation [19]:

$$ICC\;=\;\frac{\mathrm{BSMSS}\;-\;\mathrm{WSMSS}}{\mathrm{BSMSS}\;+\;\mathrm{WSMSS}}$$

where BSMSS and WSMSS are the mean sum of squares between subjects and within subjects, respectively. In the test-retest study, an ICC value ranges from −1 (no reliability) to 1 (maximum reliability) [20],[21]. Sample sizes were calculated to detect a 20-percent difference in BP_ND_ between independent groups (two-tails *t*-test) using the software G*power 3.1 [22]. The confidence level was set to be 5% (*P* < 0.05) and statistical power to 0.8. The mean of the test scans was used as the mean of baseline scans, and the SDs of the baseline and blocking scans were assumed to be same as the SDs of the test scans. All statistical parameters except for power analysis were calculated with MATLAB Version 7.12.0.635 (the MathWorks Inc., Natick, MA, USA) and Microsoft Excel 2010 (Microsoft, Redmond, WA, USA).

## Results

### Injection parameters

Mean injected radioactivity and mean specific activity at the time of injection were 687 ± 73 MBq (range: 615 to 767 MBq) and 195 ± 80 GBq μmol^−1^ (range: 131 to 305 GBq μmol^−1^), respectively, for test scans (*n* = 5) and 731 ± 53 MBq (range: 690 to 822 MBq, *n* = 5) and 143 ± 60 GBq μmol^−1^ (range: 87 to 213 GBq μmol^−1^), respectively, for retest scans (*n* = 5). The injected dose and specific activity did not significantly differ between the test and retest scans (paired *t*-test, *P* = 0.21 for injected dose and *P* = 0.24 for specific activity).

### Arterial input function

**Figure 1:**
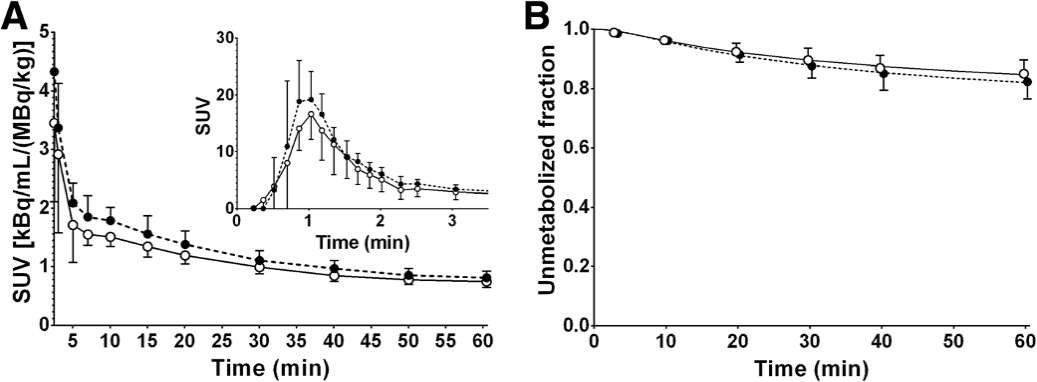
**Mean ± SD of metabolite-corrected input function and unmetabolized**^**11**^**C-TMSX fraction. (A)** Mean ± SD of metabolite-corrected input function for four healthy human subjects. The inserted graph corresponds to early data. The unit of plasma data was SUV [concentration/(injected dose/body weight)]. **(B)** Mean ± SD of unmetabolized ^11^C-TMSX fraction and mean of fitted curve for four healthy human subjects. The parent fraction was fitted using a sum of exponentials. Open and closed circles correspond to test and retest scans, respectively. Error bars show the standard deviation.

Figure 1A shows the averaged radioactivity in plasma with metabolite correction for test and retest scans (*n* = 4). The metabolism speed of ^11^C-TMSX was slow in both scans (Figure 1B): the unchanged fraction was still 85% ± 5% in test scan and 82% ± 6% in retest scan at 60 min post-injection. The free fraction of ^11^C-TMSX in plasma was 2.40% ± 0.96% for test scans and 2.40% ± 0.47% for retest scans. There are no significant differences in *f*_P_ between test and retest scans (paired *t*-test, *P* = 0.72). The ultrafiltrate-to-saline ratio was 46% ± 3% in test scans and 46% ± 3% in retest scan, indicating a high retention on the filter.

### Quantitative analysis

**Figure 2:**
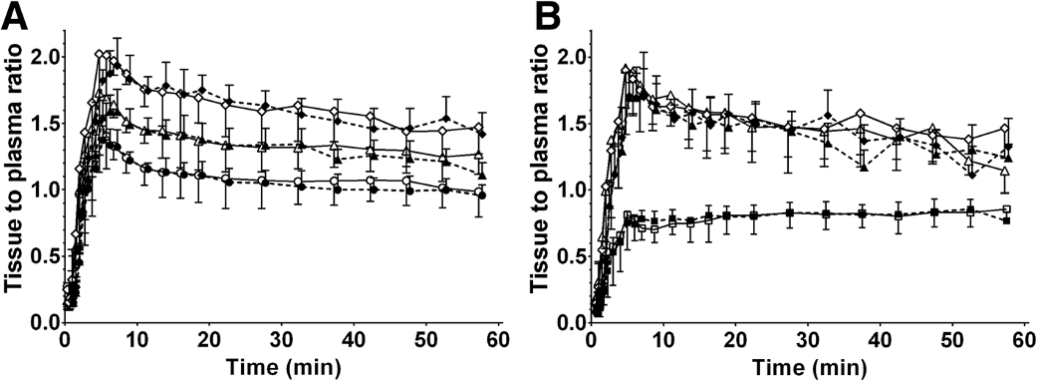
**Tissue-to-plasma ratio curve averaged across subjects (** ***n***  **= 4) in six ROIs.** ROIs are **(A)** putamen (diamonds), cerebellum (triangles), and cortex (circles) and **(B)** caudate head (diamonds), thalamus (triangles), and centrum semiovale (squares). Open symbols and closed symbols show test and retest scans, respectively. Error bars show the standard deviation.

**Figure 3:**
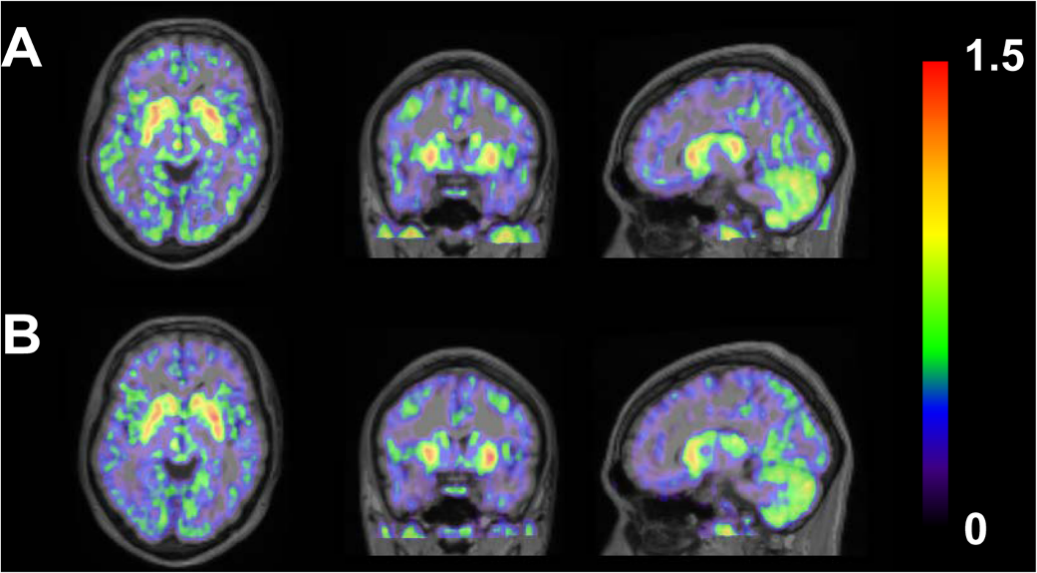
**Typical example of parametric image for BP**_**ND**_**in (A) test and (B) retest conditions.** The centrum semiovale was used as a reference region. The parametric image was fused with the individual subject’s MR image.

Brain activity in all regions reached the peak around 5 min post-injection of ^11^C-TMSX, and then gradually decreased. The average tissue-to-plasma ratio was shown in Figure 2. Typical parametric images of BP_ND_ were displayed in Figure 3 using a centrum semiovale as a reference region. The ratios in most regions became constant around 20 min post-injection of ^11^C-TMSX. The ratios in the putamen were decreased slightly throughout the scan. The values for *V*_T_ and BP_ND_ were summarized in Tables 1, 2, and 3. For each outcome parameter, the mean of the test and retest scans, the TRV (mean ± standard deviation), the aTRV, and the ICC were listed. For *V*_T_ values, the mean TRV was smaller than 5%, and always smaller than its standard deviation, indicating that there is no systematic trend between test and retest scans. The absolute TRV was ≤10% except for the thalamus. The ICC values were moderate (>0.65) except for the thalamus (0.27). We also calculated the normalized *V*_T_ (*V*_T_/*f*_P_). Global mean aTRV values were 8% and 15% for *V*_T_ and *V*_T_/*f*_P_, respectively, indicating that normalizing by the plasma free fraction *f*_p_ increased the variability of the outcome measure for ^11^C-TMSX. For BP_ND_ values, the mean TRV was between ±10% using either cerebral cortex or centrum semiovale as a reference region, and always smaller than its standard deviation, further indicating that there was no systematic trend between test and retest scans. Since BP_ND_ of the cerebellum from one subject was close to 0 in the test and retest scans with reference LGA with the cerebral cortex as a reference region, TRVs of the subject were different from the other subjects. Those values were removed from Table 2.

**Table 1:** **Test-retest variability and reproducibility of** ***V***_T_

Regions	***V***_T_[mL/cm^3^]^a^		aTRV^b^[%]	TRV^b^[%]	ICC^c^
	Test	Retest			
Putamen	1.43 (15%)	1.40 (6%)	8	1.0 ± 10	0.69
Anterior putamen	1.46 (15%)	1.44 (7%)	7	0.8 ± 10	0.68
Posterior putamen	1.39 (16%)	1.37 (6%)	10	1.2 ± 11	0.66
Caudate head	1.33 (15%)	1.26 (9%)	6	4.4 ± 7	0.77
Thalamus	1.30 (17%)	1.23 (2%)	13	4.4 ± 16	0.27
Cerebellum	1.20 (15%)	1.16 (8%)	7	2.6 ± 8	0.77
Cerebral cortex	0.98 (18%)	0.94 (9%)	7	2.8 ± 10	0.74
Centrum semiovale	0.71 (16%)	0.70 (7%)	7	0.6 ± 10	0.73

^a^Logan graphical analysis with input function (t* = 10 min) (*n* = 4). Data are presented as mean (%COV); ^b^TRV = (*p*_test_ − *p*_retest_)/(*p*_test_ + *p*_retest_) × 2, and aTRV is the absolute value of TRV; ^c^ICC = (BSMSS − WSMSS)/(BSMSS + WSMSS) where BSMSS is the mean sum of squares between subjects and WSMSS is the mean sum of squares within subjects.

**Table 2:** **Test-retest variability and reproducibility of binding potential (BP**_ND_ **) using cerebral cortex as a reference region**

Regions	Logan graphical analysis with input function (t* = 10 min,***n*** = 4)					Reference Logan graphical analysis (t* = 10 min,***n*** = 5)				
	Test^a^	Retest^a^	aTRV^b^[%]	TRV^b^[%]	ICC^c^	Test^a^	Retest^a^	aTRV^b^[%]	TRV^b^[%]	ICC^c^
Putamen	0.46 (11%)	0.49 (9%)	6	−6 ± 5	0.77	0.50 (17%)	0.53 (16%)	5	−5 ± 4	0.94
Anterior putamen	0.50 (15%)	0.52 (6%)	11	−6 ± 11	0.48	0.53 (18%)	0.57 (16%)	11	−7 ± 9	0.80
Posterior putamen	0.42 (15%)	0.45 (13%)	14	−6 ± 15	0.47	0.47 (21%)	0.48 (16%)	11	−2 ± 13	0.80
Caudate head	0.37 (23%)	0.33 (13%)	21	8 ± 26	0.18	0.38 (20%)	0.39 (29%)	19	0 ± 24	0.58
Thalamus	0.33 (8%)	0.31 (34%)	25	9 ± 31	0.38	0.33 (8%)	0.32 (27%)	22	5 ± 28	0.24
Cerebellum	0.23 (20%)	0.23 (14%)	15	−1 ± 22	0.44	0.23 (20%)^d^	0.23 (14%)^d^	14^d^	**−** 2 ± 22^d^	0.47^d^

^a^Data are presented as mean (%COV); ^b^TRV = (*p*_test_ − *p*_retest_)/(*p*_test_ + *p*_retest_) × 2, and aTRV is the absolute value of TRV; ^c^ICC = (BSMSS − WSMSS)/(BSMSS + WSMSS) where BSMSS is the mean sum of squares between subjects and WSMSS is the mean sum of squares within subjects; ^d^Values for the cerebellum were calculated from four subjects.

**Table 3:** **Test-retest variability and reproducibility of binding potential (BP**_ND_ **) using centrum semiovale as a reference region**

Regions	Logan graphical analysis with input function (t* = 10 min,***n*** = 4)					Reference Logan graphical analysis (t* = 10 min,***n*** = 5)				
	Test^a^	Retest^a^	aTRV^b^[%]	TRV^b^[%]	ICC^c^	Test^a^	Retest^a^	aTRV^b^[%]	TRV^b^[%]	ICC^c^
Putamen	1.02 (6%)	1.01 (4%)	1	1 ± 2	0.90	1.06 (9%)	1.03 (7%)	3	2 ± 4	0.87
Anterior putamen	1.07 (4%)	1.06 (3%)	4	1 ± 6	−0.10	1.10 (9%)	1.09 (4%)	5	1 ± 6	0.69
Posterior putamen	0.97 (12%)	0.96 (7%)	6	1 ± 10	0.53	1.02 (12%)	0.97 (11%)	7	5 ± 8	0.68
Caudate head	0.89 (14%)	0.80 (5%)	13	10 ± 14	0.00	0.90 (17%)	0.85 (2%)	13	4 ± 17	0.11
Thalamus	0.84 (6%)	0.77 (14%)	14	9 ± 17	−0.37	0.83 (22%)	0.77 (19%)	15	7 ± 17	0.65
Cerebellum	0.70 (9%)	0.65 (2%)	8	6 ± 9	0.05	0.63 (42%)	0.58 (41%)	10	8 ± 9	0.95

^a^Data are presented as mean (%COV); ^b^TRV = (*p*_test_ − *p*_retest_)/(*p*_test_ + *p*_retest_) × 2, and aTRV is the absolute value of TRV; ^c^ICC = (BSMSS − WSMSS)/(BSMSS + WSMSS) where BSMSS is the mean sum of squares between subjects and WSMSS is the mean sum of squares within subjects.

The mean BP_ND_ values were larger, and the TRV and aTRV were smaller when using the centrum semiovale as reference instead of the cerebral cortex. Global mean aTRV values were 8% and 15% for BP_ND_ using the centrum semiovale and cerebral cortex, respectively, as a reference region. Both TRVs using LGA with input function were comparable to those values using reference LGA. The BP_ND_ estimates using LGA with input function were in excellent agreement with those from reference LGA (BP_ND, reference LGA_ *=* 1.00 BP_ND, LGA_ + 0.01, *R*^2^ = 1.00 with the cerebral cortex as a reference region, BP_ND, reference LGA_ *=* 1.05 BP_ND, LGA_ + 0.01, *R*^2^ = 0.98 with the centrum semiovale).

Power analysis (two-tails *t*-test, statistical power 0.8) was conducted to estimate the samples sizes to detect a 20-percent difference in BP_ND_. Using the cerebral cortex as a reference region, sample sizes ranged from 4 (thalamus) to 21 (caudate head) per group. Using the centrum semiovale as a reference region reduced the required sample sizes: 3 (putamen and thalamus) to 9 (caudate head).

## Discussion

The plasma free fraction (*f*_P_) was measured in this study, allowing for correction of *V*_T_ values. This correction by *f*_P_ is useful if *f*_P_ can be measured reliably and if there is substantial intra-subject variation. In our measurements, the *f*_P_ was consistently low (<3%), with evidence that ^11^C-TMSX stuck to the ultrafiltration tubes, which may lead to underestimation of *f*_p_. However, the *f*_P_ value measured in [23] was 9.1% ± 0.4% (*n* = 6, human) by the ultrafiltration method. Such a discrepancy might be attributable to high stick factor in our data. Note that the stick factor was not reported in [23]. Another possibility is the difference in the preparation of the injection solution. Finally, the signed and absolute TRVs were larger for *V*_T_/*f*_P_ compared to those of *V*_T_. Hence, the normalization of *V*_T_ by *f*_P_ did not reduce variability in this case.

Inter-subject variability (% coefficient of variation (COV)) of *V*_T_ at retest scans were lower (approximately 7%) than that of the test scans (approximately 16%), while no significant difference was observed in the injected dose, specific activity, and *f*_P_ of ^11^C-TMSX. Another possibility to explain the difference in the inter-subject variability is a difference in the metabolism of the tracer. The subjects were controlled for caffeine intake, but not for smoking habituations. Nicotine consumption might change the metabolism of ^11^C-TMSX as seen in the study with adenosine A_1_ receptor ligand ^18^ F-CPFPX [24]. In a retrospective investigation, it turned out that subjects consisted of a nonsmoker, a smoker (blood sampling was not available), and three subjects with unknown status. However, the parent fraction of ^11^C-TMSX was very high and well reproducible (Figure 1B). Therefore, we concluded that a change in the metabolism speed was not a reason to increase the inter-subject variability. In contrast to *V*_T_, such a difference in the inter-subject variability did not exist in BP_ND_. The difference in the %COV between test and retest scans might come from errors included in the input function measurement.

The test-retest variability and reliability of *V*_T_ were good (aTRV ≤10%, ICC >0.6) across regions except for the thalamus (aTRV: 13% and ICC: 0.27). For BP_ND_, a good absolute TRV was seen in the high A_2A_ regions (putamen and caudate). However, lower-binding regions (BP_ND_ < 0.4) showed high aTRV (>15%) and low ICC values; this is not surprising, since BP_ND_ is small in those regions. We examined the test-retest variability data of *V*_T_ and BP_ND_ from a number of radioligands. The aTRV of *V*_T_ of ^11^C-TMSX (8% averaged across all regions) was comparable to that of other radioligands used to study dopamine and adenosine receptors. The reported aTRV values of *V*_T_ were 5% to 11% (average: 7%) with ^11^C-FLB457 [25] for dopamine D_2/3_ receptor and 12% to 14% (average: 13%) with ^18^ F-CPFPX [26] for adenosine A_1_ receptor. The aTRV of BP_ND_ with ^11^C-TMSX was comparable to that with ^11^C-FLB457 (6% to 15%) and larger than that with ^18^ F-FPFPX (3% to 9%).

Given the good reproducibility of *V*_T_, ^11^C-TMSX should be suitable for use in receptor occupancy studies with input function. The range of *V*_T_ values was not wide across regions (0.70 to 1.46 mL/cm^3^). However, using the occupancy plot [27] is feasible using the regions with a narrow range of *V*_T_ values with ^11^C-GSK931145 for glycine type 1 transporter (0.43 to 0.79 mL/cm^3^) [28] and ^18^ F-CPFPX for adenosine A_1_ receptor (0.42 to 0.82 mL/cm^3^) [29]. Note that the occupancy plot assumes that the receptor occupancies are uniform in all regions of interest. Previous reports [1],[30] suggest that some regions might have an ‘atypical’ binding. Therefore, we need to carefully choose regions used for the occupancy plot with ^11^C-TMSX. Another possible way for estimating receptor occupancy is to estimate a relationship between blocking dose (or plasma level) and *V*_T_ for each region [31]. This second method can be used even if all regions have the same baseline *V*_T_.

The test-retest variability of BP_ND_ values using the cerebral cortex as a reference region was larger than those using the centrum semiovale. In the striatum, the high A_2A_R-binding region, the aTRVs of BP_ND_ were 5% in the putamen and 19% in the caudate head using the cerebral cortex as a reference region. On the other hand, the aTRVs of BP_ND_ were 3% in the putamen and 13% in the caudate head using the centrum semiovale as a reference region. This is partly because the BP_ND_ value was smaller using the cerebral cortex as a reference region.

The thalamus showed a low reproducibility of both *V*_T_ and BP_ND_ values. Moreover, while the mean distribution volume in the thalamus was high, a *postmortem* study with ^3^H-SCH58261 [15] showed that A_2A_R density is low. The uptake in the thalamus is considered to be ‘atypical’ binding [1],[30], which is different from classical A_2A_R binding. This low reproducibility in the thalamus may be partly due to such an ‘atypical’ binding. Thus, given the low reproducibility and ‘atypical’ binding, the thalamus should be carefully considered in further clinical research.

Using either the cerebral cortex or centrum semiovale as a reference region, reference LGA and LGA with input function provided similar BP_ND_ values. The TRV and aTRV of BP_ND_ were slightly smaller using the reference LGA. Not surprisingly, the reference tissue model is not affected by errors in the measurement of input function. This suggests that the reference LGA can be useful for further studies.

There are two limitations of this study: unknown optimal reference region for ^11^C-TMSX and small sample size. As far as we know, the only available A_2A_R blocking study using an antagonist radiotracer *in vivo* in human brain is a ^11^C-KW-6002 PET study with varying dose of cold KW-6002 [3] However, blocking results in the centrum semiovale and neocortical regions were not included in the report. Thus, the suitability of the cerebral cortex or central semiovale as a reference region has yet to be determined by blocking or occupancy studies. Due to a lack of blocking study and *postmortem* study in the regions with low A_2A_R density, the region with lowest *V*_T_ was chosen. For the SPECT A_2A_R tracer ^123^I-MNI-420, while a reference region is not yet validated, a test-retest reproducibility of BP_ND_ was evaluated to facilitate the comparison between ^123^I-MNI-420 and other A_2A_R radiotracers [32]. We also took an exploratory approach to calculate BP_ND_ values using candidate reference regions in order to evaluate BP_ND_ reproducibility. However, the determination of the reference region is most desirable in order to establish the utility of ^11^C-TMSX for PET imaging. In this study, we evaluated outcome measures with input function in four subjects. We examined sample sizes for test-retest human studies using other radioligands. As far as we know, the minimum sample size is three subjects for test-retest protocol (^18^ F-MK-6577 [33] for glycine transporter type 1 and ^123^I-MNI-420 [32] for A_2A_R).

## Conclusions

The quantification of ^11^C-TMSX imaging was reproducible for PET studies of A_2A_R. The LGA with input function achieved good reproducibility for *V*_T_ in all regions. The results support the use of PET and ^11^C-TMSX as a suitable tool for receptor occupancy studies. The use of the cerebral cortex or centrum semiovale as a reference region with invasive and reference LGA produced good or moderate reproducibility of the BP_ND_ in high A_2A_R regions. While the centrum semiovale showed higher reproducibility of the BP_ND_, blocking studies are required to determine the optimal reference region conclusively.

## Authors’ original submitted files for images

Below are the links to the authors’ original submitted files for images. Authors’ original file for figure 1 Authors’ original file for figure 2 Authors’ original file for figure 3

## Abbreviations

^11^C-TMSX: [7-methyl-^11^C]-(*E*)-8-(3,4,5-trimethoxystyryl)-1,3,7-trimethylxanthine
A_2A_R: adenosine A_2A_ receptor
aTRV: absolute test-retest variability
BP_ND_: binding potential relative to non-displaceable uptake
DVR: distribution volume ratio
*f*_P_: plasma free fraction
HPLC: high-performance liquid chromatography
ICC: intra-class correlation coefficient
*V*_T_: distribution volume
TACs: time-activity curves
TRV: signed test-retest variability
