# Supplementary figures and images for: Test-retest variability of adenosine A2A binding in the human brain with 11C-TMSX and PET
Source: EJNMMI Res. 2014 Dec 29;4:76. doi: 10.1186/s13550-014-0076-9 (PMC4293456; doi:10.1186/s13550-014-0076-9)

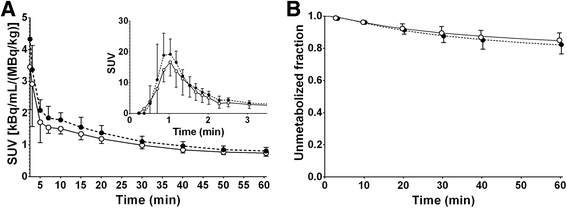

Supplement: Supplementary file 1 — Authors’ original file for figure 1 [file 13550_2014_76_MOESM1_ESM.gif]

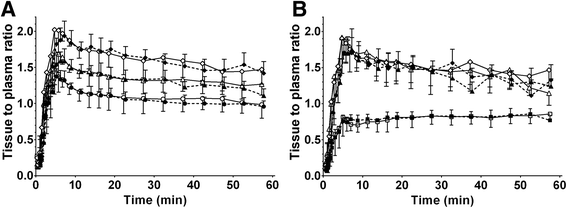

Supplement: Supplementary file 2 — Authors’ original file for figure 2 [file 13550_2014_76_MOESM2_ESM.gif]

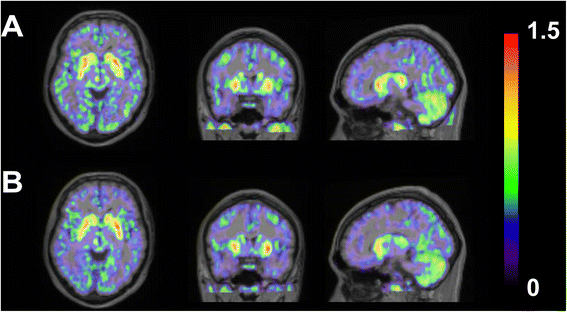

Supplement: Supplementary file 3 — Authors’ original file for figure 3 [file 13550_2014_76_MOESM3_ESM.gif]
